# Mindfulness training combined with cold water immersion effects on mood and perception of executive functioning in middle-aged and older adults: a pilot study

**DOI:** 10.3389/fpubh.2025.1693026

**Published:** 2025-11-07

**Authors:** Ambra Gentile, Sara Vivirito, Musa Kirkar, Konstantinos Paschos, Luka Tuđan, Jakub Kulhánek, Pelin Öztürk, Marianna Alesi

**Affiliations:** 1 Department of Psychology, Educational Sciences and Human Movement, University of Palermo, Palermo, Italy; 2 CEIPES ETS, Palermo, Italy; 3 European Network of Active Living for Mental Health, Brussels, Belgium; 4 Swimming Club Zagreb, Zagreb, Croatia; 5 Shut Up and Swim, Prague, Czechia; 6 Innovative Education Center, Vienna, Austria

**Keywords:** healthy aging, older adult, mindfulness, middle-aged adults, cold water

## Abstract

**Background:**

Age-related cognitive decline typically begins during middle age and persists into old age. In parallel, mood (and, in particular, anxiety and depression) can be a significant predictor of neurodegenerative pathologies. To prevent these negative consequences, mindfulness trainings were used to improve mood and executive functioning in middle-aged and older adults. Less is known about cold water immersion, which apparently favors cognitive enhancement and mood restoration.

**Methods:**

The current pilot study involved a sample of 46 adult participants (63% F; mean age: 60.67 years, SD: ±8.51), who completed a combined mindfulness and cold-water immersion program of 20 weeks. Before and after the training period, participants completed questionnaires about depressive and anxiety symptoms and executive functioning perception. A linear mixed model was run to evaluate pre-post differences with the presence of potential confounders (i.e., country, occupation, physical activity practice).

**Results:**

The results showed a positive effect of the combined training on depression (mean difference = −2.59, *t* = −3.10, *p* = 0.003), with higher effectiveness for older adults compared to middle-aged participants (mean difference = −3.26, *p* = 0.042). Moreover, a significant effect of the training was found concerning anxiety (F_1,50.08_ = 7.70, *p* = 0.008), without differences between the two age groups (F_1,51.06_ = 0.10, *p* = 0.75). Finally, a non-significant effect of the combined training was found between pre- and post-treatment phase concerning executive functioning perception (F_1,52.64_ = 3.61, *p* = 0.06).

**Conclusion:**

Cold water immersion combined with mindfulness sessions could be considered by future researchers as a possible training for healthy aging.

## 1 Introduction

The global population is undoubtedly aging, posing significant challenges for the healthcare system (1). Therefore, there is an increasing need to identify strategies for promoting healthy lifestyle starting with middle-aged adults (2).

There is a large consensus that cognitive decline starts from 60 years (3), although some studies indicate a earlier cognitive decline declining (4). For example, a study of Ferreira et al. (5) reported that executive functioning decline may start from the age of 50. Executive functioning are higher-ordered cognitive functions supporting goal-directed behaviors (6). According to the model of Diamond (7), the three main core executive functions are inhibitory control, working memory and cognitive flexibility. Inhibitory control allows the suppression of irrelevant stimuli in favour of a relevant one, or the ability to control one’s behavior, while working memory is the ability to keep in mind and manipulate information no longer present in the environment. Cognitive flexibility refers to the ability to switch between perspectives or maintain multiple perspectives simultaneously (8). Executive functions are deeply implicated in movement, and several studies have shown their role in predicting falls in healthy older adults (9–11).

Additionally, mood regulation may vary throughout the lifespan (12). Despite a higher prevalence of psychiatric disorders in late life (13), scientific literature generally agrees on older adults’ effective mood regulation (14), with a reduction trend of depressive symptoms with age (13). Indeed, some studies highlighted that depressive and anxiety symptoms are associated with dementia risk (14–16). Moreover, Gimson et al. (17) reported that midlife anxiety is associated with an increased risk for late-life dementia.

According to these studies, the protection of executive functions and mood balance in middle-aged and older adults might reduce the burden on the public health system. Indeed, on the one hand, risk of falls might be reduced by enhancing executive functions. On the other hand, lowering anxiety and depression symptoms might also contribute to reducing dementia risk.

For these purposes, scientific literature indicates mindfulness training as an effective program for enhancing executive functioning (18). In recent years, mindfulness has become one of the primary approaches for enhancing mental wellbeing (19–21). In particular, the original mindfulness program created by Kabat-Zinn (22) focuses on individual, non-judgmental awareness of the present moment, eliciting positive emotions and increasing attentional focus (23). Moreover, scientific literature has also indicated cold water immersion as a tool for improving mood (24). Kunutsor et al. (25) highlighted the beneficial effects of cold-water immersions as a good practice for healthy aging. In their review, the Authors found psychological beneficial effects in terms of executive function and neurodegenerative disease protection, since the exposure to cold water stimulates the production of norepinephrine, a neurotransmitter connected to attention and mood. Specifically, optimal levels of norepinephrine are connected to better executive functioning and reduced distractibility (26), and a better mood regulation (27).

Considering the beneficial effects, respectively, of mindfulness training and cold-water immersion on mood regulation and executive functioning, the combination of these two approaches may yield synergistic positive effects on middle-aged and older adults’ psychological wellbeing. In particular, both trainings might separatedly determine a change at neurological level stimulating the production of neurotransmitters and improving attentional focus, a combined training might show complementary neurobiological effects. As previously affirmed, cold water stimulates the production of norepinephrine (25). Similarly, Craigmyle (28) found that mindfulness mediation practices, such as mind-wandering, activates the anterior cingulate cortex, which modulates the locus coerelus, increasing the levels of norepinephrine.

Concerning previous studies, Faid et al. (29) in the only one combining cold water immersion and meditation with the Wim-Hoff method, which integrates breathing exercises and meditation in a cold environment (30), whilst no studies were found on middle-aged and older adults.

Middle-aged and older adult populations represent different phases of the aging process and might be characterized by changes at different stages. The comparison between these two groups might offer the possibility to identify age-specific effects of the intervention. Previous literature reported that older adults tend to have a better mood regulation than middle-aged adults (14), who are in the peak years of work career and familiar responsibilities. Therefore, it is expected a higher effectiveness of the training on middle-aged adults compared to older adults, that already have a good mood regulation. Conversely, concerning executive functioning, older adults might experience cognitive decline in a higher extent than middle-aged adults (31, 32). Thus, in this case, it is expected that the program would be more effective for older adults compared to middle-aged.

Therefore, given the importance of preserving executive functioning and mood balance starting from middle-aged adults, the aim of the current pilot study is to analyse the effect of a mindfulness program combined with cold water immersion in a sample of middle-aged and older adults. Specifically, the study hypothesizes that:

*H1*: the program would have beneficial effects on the overall sample on depressive and anxiety symptoms;

*H2*: the program would have more positive effects on the mood of middle-aged adults;

*H3*: the program would have more positive effects on executive functioning perception of older adults.

## 2 Methods

### 2.1 Participants

For requirements Participants were recruited through public announcements in different countries (Italy, Croatia, Austria, Belgium, Czechia) voluntarily.

The recruitment was part of the COL.D.D. project—*Cold water swimming for the prevention of Dementia and Depression* (Project: 101133918—COL.D.D.—ERASMUS-SPORT-2023). The project was developed within an international consortium composed of CEIPES ETS (Italy), the European Network of Active Living for Mental Health (Belgium), Swimming Club Zagreb (Croatia), SHUT UP AND SWIM (Czechia), and the Innovative Education Center (Austria), with the University of Palermo involved as an associated academic partner.

The inclusion criteria for the participation to the program were being healthy, without pathologies, not taking medication and being >50 years. Therefore, participants with pathologies, taking medication or younger than 50 years were excluded.

Sample size was estimated through G*Power for multiple regression model, with an effect of 0.35 [taken from Faid et al. (29)], 80% of power and 5 predictors, resulting in 43 participants. The initial sample consisted of 54 participants; however, 6 participants withdrew from the procedure at different stages of the intervention, while 2 participants did not complete the post-intervention evaluation. Therefore, the final sample consisted of 46 participants (63% F), whose mean age was 60.67 years (Standard Deviation [SD]: ±8.51). More than half of the sample was married (52%), living in different parts of Europe (Austria: 22%, Belgium: 17%, Croatia: 13%, Czechia: 26%, Italy: 22%), and were mainly retired (65%).

In the pre-intervention phase, participants were reunited in a room for explaining the purpose of program, the required time effort, and the physician explained benefits and risk connected to the procedure. Participants were also informed to be free to withdraw from the procedure at any stage and without consequences. Afterwards, those willing to participate to the intervention were asked to sign a written informed consent. Therefore, the researcher gave to each participant a battery of questionnaires about perception of executive functioning, anxiety and depressive symptoms. After the intervention, participants were asked to complete again the questionnaires. The training lasted 3 months, from November 2024 to March 2025, twice a week for 1 h, for a total of 40 training sessions. All the participants followed all the training sessions.

The study was conducted respecting the Declaration of Helsinki principles and was approved by the Bioethical Committee of the University of Palermo, protocol nr. 248/2024 of the 26/11/2024.

### 2.2 Measures

#### 2.2.1 Depressive symptoms

To assess the presence and the level of depressive symptoms, the adapted version of the Beck Depression Inventory Short-Form (BDI-SF) (33) was employed. The original scale is one of the most used worldwide to screen depressive symptoms, while the short form revealed to be effective especially for detecting depression in older adults (34). The SF consists of 13 items rated on a 4-point Likert scale ranging from 0 to 3, with higher scores indicating higher rates of depression. Participants are asked to evaluate their feelings in the past 2 weeks and to endorse the sentence that is nearer to their experience (0: “I do not feel sad”; 1: “I feel sad”; 2: I am sad all the time and I cannot snap out of it”; 3: I am so sad or unhappy that I cannot stand it”). The scale showed an excellent internal consistency (Cronbach alpha: 0.96).

#### 2.2.2 State anxiety

State anxiety was detected through the state anxiety subscale of the Spielberger State–Trait Anxiety Short Form (STAI SF) (35). The scale consists of two measures of anxiety, namely trait anxiety and state anxiety. For the purposes of the study, state anxiety only was taken in consideration, as we hypothesize that the training is effective on situational anxiety. The subscale consists of 5 items evaluated on a 4-point Likert scale (sample item: “I feel that difficulties are piling up so that I cannot overcome them”), ranging from 1 (“Not at all”) to 4 (“Very much so”). Higher scores indicate higher levels of anxiety. The internal consistency of the scale was good (Cronbach alpha: 0.81).

#### 2.2.3 Executive functioning perception

Executive functioning perception was assessed through the Amsterdam Executive Function Inventory (AEFI) (36), which is a self-reported measure of executive functioning. It consists of 13 items measured on a 3-point Likert scale (1: “Not true,” 2: “Partly true,” 3: “true”). The items are grouped into three subscales, namely attention, planning and self-control (sample item: “my thoughts easily wander”). For the purposes of the study, we considered the general score, obtained through the raw sum of all the items. The internal consistency of the scale was 0.54, which is in line with the original standardization of the questionnaire (36).

#### 2.2.4 Mindfulness training combined with cold water immersion

Participants were recruited in each Consortium country (Italy, Belgium, Croatia, Czechia, and Austria) and attended forty workshops twice a week, from November 2024 to March 2025. Each group session, lasting about 2 h, comprised the combination between mindfulness training and cold-water immersion. The whole training was supervised by a physician in case of side effects.

Each session was conducted by a trainer who previously followed a transnational intensive training course in Croatia in July 2024. This training course had the purpose to instruct the trainers about the project and to deepen the theoretical rationale of the training. Moreover, trainers received information about how conducting safely the cold-water immersions and the mindfulness training.

The mindfulness training was delivered by psychologists certified with mindfulness courses or diploma. Each of the 40 sessions consisted of two phases: initially, 40 min were dedicated to mindfulness techniques, starting from the present moment awareness (about 10 min). Then, the body scan technique was performed together with progressive muscle relaxation (about 15 min). Finally, the training ended with guided breathing exercises (about 10 min). At the end of the session, 5 min were dedicated to open discussion (5 min), where participants were encouraged to share their feedback about the training session, and to compare the present experience to the past ones. Participants were also asked to repeat these techniques individually at home between training sessions.

After the mindfulness session, participants underwent to cold water immersions, conducted in controlled environments, with a special focus on safety procedures. Participants were initially required to take a cold bath in standing position for 1 min with a temperature of 14 °C, which was lowered session to session as participants increased their tolerance. Participants were immersed from their feet up to their shoulders. At the end of the program, participants were able to stay immersed in 8 °C for 20 min maximum. No side effects were encountered during the whole program.

### 2.3 Data analysis

Data were analyzed through the software R (version 4.4.1.) with lmerTest package. Descriptive statistics is presented in Table 1. Considering that there studies generally indicate that executive functioning decline can start from 60 years (3), participants were divided into middle aged adults (from 45 to 59 years) and older adults (>60 years). Pre-test and post-test differences were evaluated through a linear mixed model with confounders (physical activity practice, occupational status, and country). Specifically, the model included the following variables as fixed effects: phase (pre- vs. post-test), age group (middle-aged vs. older adults), physical activity practice (sedentary vs. active), occupational status (employed vs. unoccupied/retired), and country (Austria, Belgium, Croatia, Czechia, Italy). Within-subject variability was accounted through a random slope for each participant. Restricted Maximum Likelihood (REML) was used as estimation method.

**Table 1:** Descriptive statistics.

Variable	Pre-test						Post-test						Pre-post comparison	Cohen’s d (95% CI)
	Total Sample		Middle-aged adults		Older adults		Total sample		Middle-aged adults		Older adults			
	*M*	SD	*M*	SD	*M*	SD	*M*	SD	*M*	SD	*M*	SD	T (95% CI)	
Age	60.67	8.51	55.90	5.23	69.63	5.78								
Depression	10.48	11.22	14.73	11.34	2.50	5.01	7.35	9.06	10.37	9.71	1.69	3.40	3.95*** (1.53–4.73)	0.58 (0.27–0.89)
State Anxiety	10.28	3.26	11.30	3.22	8.38	2.42	9.09	3.76	10.20	4.08	7.00	1.75	2.88** (0.36–2.03)	0.42 (0.12–0.72)
Executive functioning	27.63	4.11	27.50	3.87	27.88	4.66	29.41	4.58	29.80	5.32	28.69	2.73	−2.40* (−3.28; −0.29)	−0.35 (−0.65; −0.05)

***p < 0.0001; **p < 0.001; *p < 0.05.

## 3 Results

### 3.1 Descriptive statistics

Significant differences emerged between the pre-test and post-test conditions concerning all variables. Specifically, there was a reduction in depression (*t* = 3.95, df = 45, 95% CI: 1.53–4.73, *p* < 0.01) and anxiety (*t* = 2.88, df = 45, 95% CI: 0.35–2.03, *p* < 0.01) symptoms, and an increase in executive functioning perception (*t* = −2.40, df = 45, 95% CI: 0.29–3.28, *p* < 0.05).

### 3.2 Differences between middle-aged and older adults

**Figure 1:**
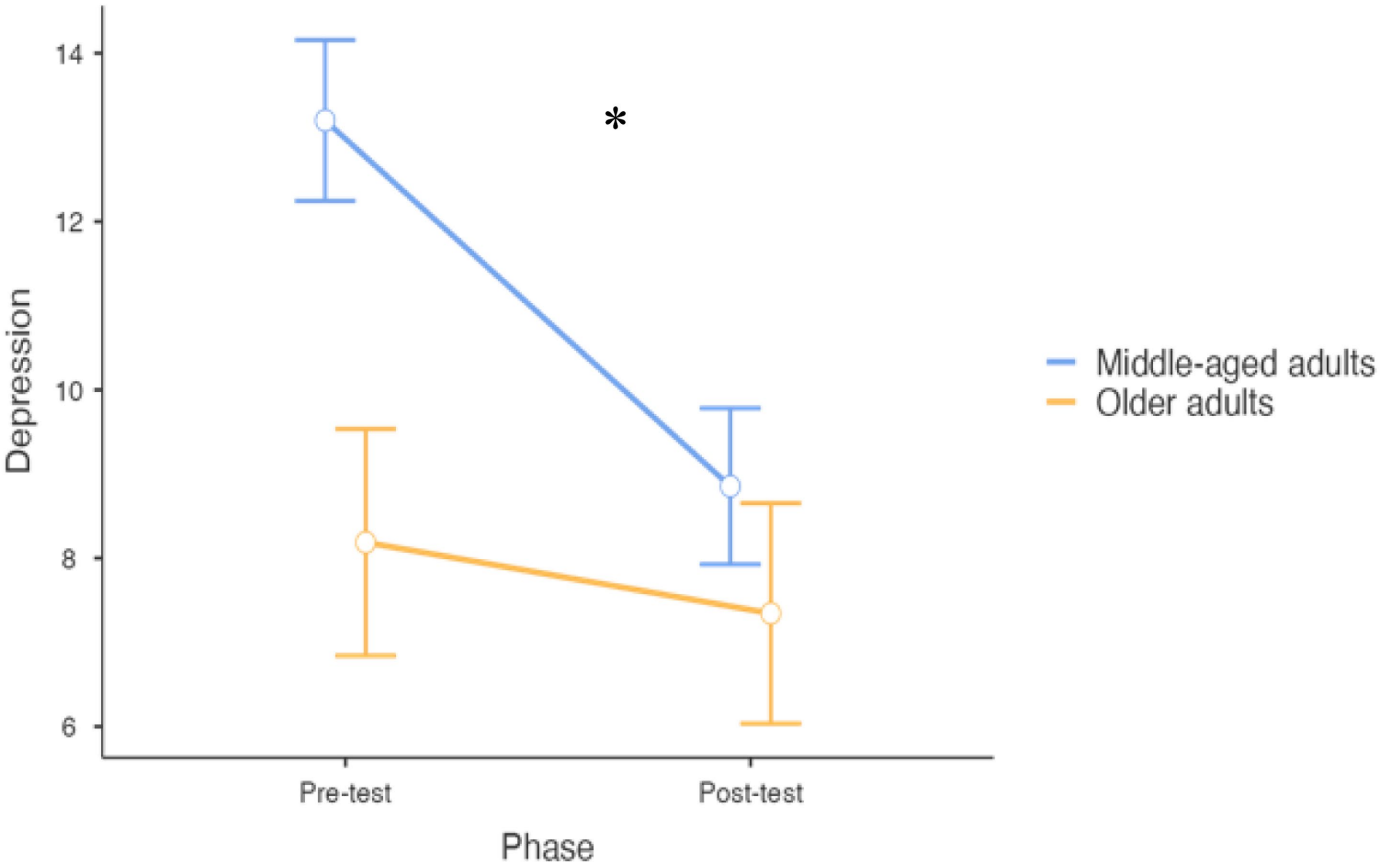
Differences between pre- and post-treatment concerning depression (error bars represent standard errors). ^*^p < 0.05.

Concerning Depression, the model reported a significant main effect of Phase (F_1,54_ = 9.39, df = 54, 95% CI: −4.26 to −0.93, *p* = 0.003), with a general post-intervention reduction (mean difference = −2.59, *t* = −3.10, df = 54, 95% CI: 0.84–4.35, *p* = 0.003) (see Figure 1). Moreover, a significant interaction between phase and age group emerged (F_1,54.17_ = 3.50, df = 54.17, 95% CI: 0.17–6.84, *p* = 0.04), highlighting that the training was more effective for older adults compared to middle-aged participants (mean difference = −3.26, df = 54, 95% CI: −4.35 to −0.84, *p* = 0.042). In addition, unoccupied/retired participants reported higher depression levels that employed participants (mean difference = −2.97, df = 49.10, 95% CI: −5.75 to −0.19, *p* = 0.039). Country had a significant effect (F_4,69_ = 69.71, *p* < 0.001) with Austria reporting the highest level of depressive symptoms, while no effects was encountered on being sedentary/active. The model accounted for the 86% of the variability concerning Depression.

#### 3.2.1 Anxiety

**Figure 2:**
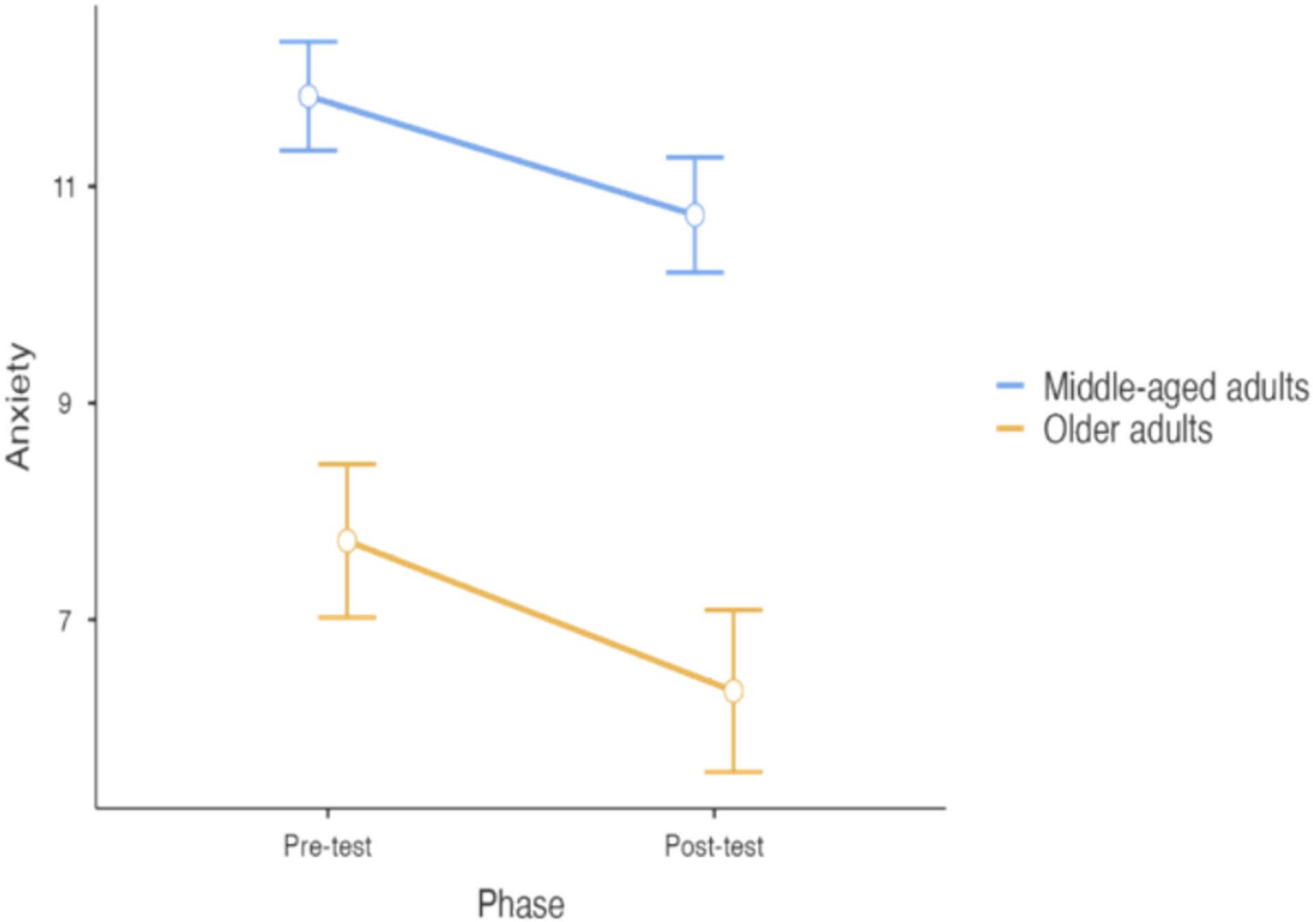
Differences between pre- and post-treatment concerning anxiety (error bars represent standard errors).

In relation to Anxiety, the model reported a significant effect of Phase (F_1,50.08_ = 7.70, *p* = 0.008), with a significant decrease in the post-test evaluation (mean difference: −1.24, t = −2.78, df = 50, 95% CI: −2.13 to −0.35, *p* = 0.008) (see Figure 2). The interaction between phase and age range was nonsignificant (F_1,51.06_ = 0.10, *p* = 0.75), indicating that this decrease was similar in the two age groups. Regarding confounders, a significant main effect of occupation (F_1,46.13_ = 7.04, *p* = 0.011) and country (F_4,49.63_ = 19.52, *p* < 0.001) were encountered. Specifically, people having a job reported higher anxiety symptoms than retired/unoccupied participants (mean difference: −2.05, df = 46.13, 95% CI: −3.58 to −0.51, t = −2.65, *p* = 0.011) and people from Austria reported the highest anxiety levels. No differences concerning people physically active or sedentary were found. The model accounted for the 66% of the variance on Anxiety.

#### 3.2.2 Executive functioning perception

**Figure 3:**
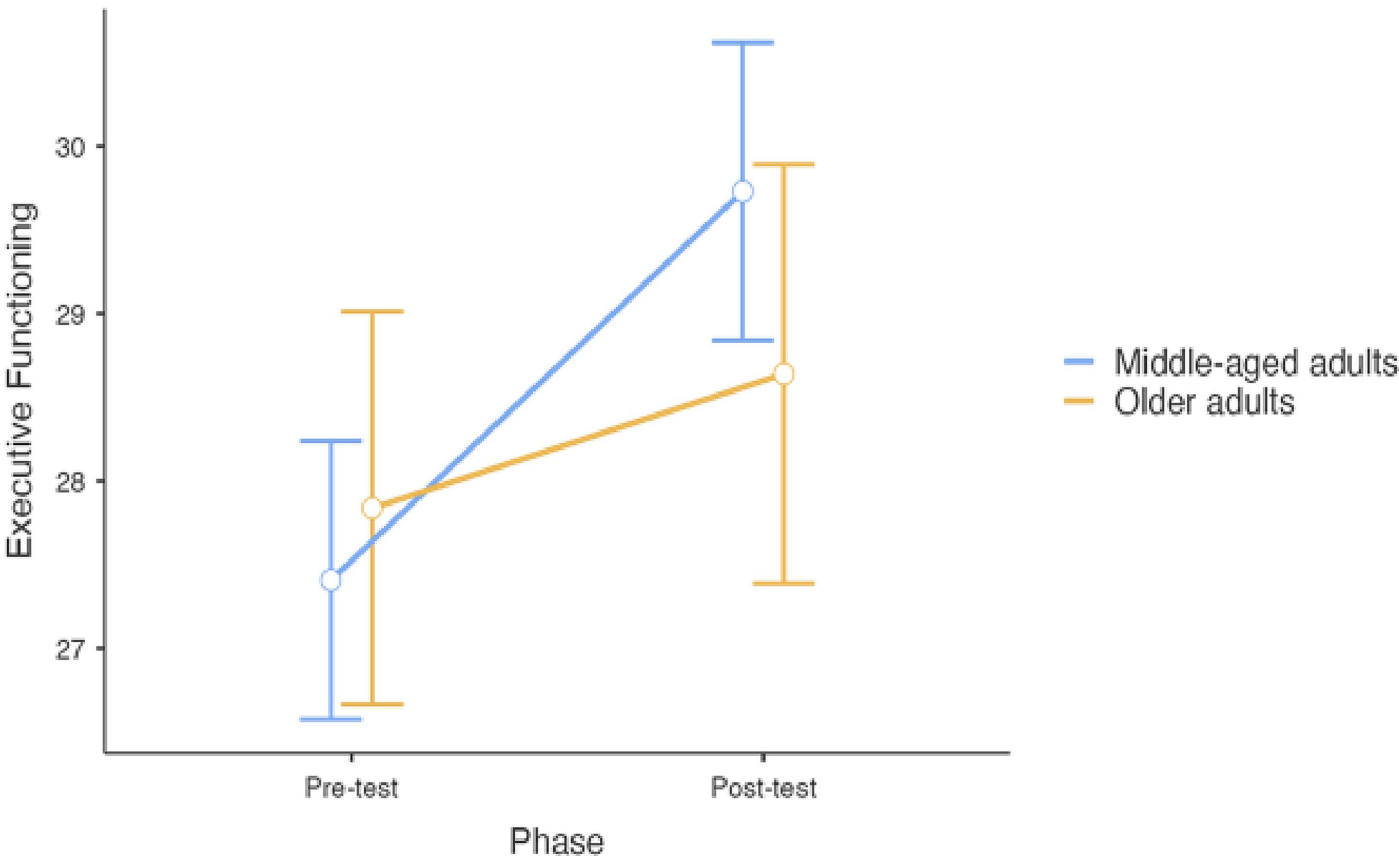
Differences between pre- and post-treatment concerning perception of executive functioning (error bars represent standard errors).

Concerning Executive Functioning perception, a non-significant effect of the combined training was found between pre- and post-treatment phase (F_1,52.64_ = 3.61, *p* = 0.06mean difference: 1.56, t = 1.90, df = 52.64, 95% CI: −0.07–3.20, *p* = 0.06) (Figure 3). No effects of age range (F_1, 49.50_ = 0.06, *p* = 0.81), physical activity practice (F_1,55.37_ = 0.99, *p* = 0.323) and occupational status (F_1, 50.61_ = 0.00, *p* = 0.987) were retrieved. Participants’ scores differed only on Country, where people from Austria reported higher executive functioning perception than people from Belgium and Czechia. The model explained a total of 29% of variance.

## 4 Discussion

The current pilot study aimed to test the effectiveness of mindfulness training combined with cold-water immersions in a sample of middle-aged and older adults. In particular, the study hypothesized that the program would have beneficial effects on the overall sample in terms of depressive and anxiety symptoms, and that the training would be more effective for improving for enhancing mood in middle-aged individuals and for improving executive functioning in older adults. The results of the study partially supported the first hypothesis and fully supported the second hypothesis, while the third hypothesis was not supported. Specifically, we found a decrease in anxiety and depressive symptoms after the intervention, with a higher effect for depressive symptoms among middle-aged adults, while the effects of the training on executive functioning were not significant.

Our results showed a decrease in anxiety and depressive symptoms which aligns with the literature on mindfulness training (33, 34) and cold water immersion. Moreover, the study is in line with Furley et al. (3), finding a significant effect of the combination between mindfulness exercises and cold water immersion.

A non-significant result was found on executive functioning, probably due to the small sample size and that we assessed the perception of executive functioning through self-reported measure. Concerning mindfulness training, its effectiveness on executive functions relies on the fact that brain plasticity adapts to change in consciousness, as demonstrated by neuroimaging techniques (37). Moreover, training the attentional focus for extended periods can modify brain electrical activity, leading to an improvement in executive functioning (38). Regarding mood improvement, the awareness of bodily states, thoughts and consciousness, and the attitude of openness and acceptance may encourage the attainment of self-regulation, reducing anxiety and depression risk (19, 39).

Similarly, a study by FitzGibbon et al. (40) measured EEG in individuals with modern hypothermia and observed a decrease in alpha activity, associated with a relaxed state, and an increase in beta activity, linked to executive functioning. Cold water exposure is also associated with a decrease in cortisol secretion as a response to acute stress (41), while repeated cold water exposure may be associated with improved stress responses, which can be generalized to enhanced mood regulation and anxiety management (41, 42).

The second result reported an overall improvement of mood regulation, and in particular, middle-aged adults’ depressive symptoms seemed to benefit the most. Previous studies have showed that older adults usually display better mood regulation strategies compared to younger people (43). Therefore, it seems that the combination of mindfulness training and cold-water immersion might be effective for individuals who are less emotionally regulated.

To our knowledge, this is the first pilot study combining mindfulness training and cold-water immersions, specifically targeting middle-aged and older adults. The advantage of this study relies on the creation of an accessible and cost-effective intervention that might increase executive functioning and mood regulation. Despite these encouraging results, the study comes out with some limitations: first, since it was a pilot initiative, no control group nor single training condition (i.e., only water immersion/only mindfulness training) were involved, therefore we cannot conclude if the training was effective thanks to the combination of the two aspects or if one of the two components was more effective. Moreover, the sample was small and unbalanced: indeed, the sample consisted predominantly of middle-aged adults, which could explain the nonsignificant results of the second and third hypotheses. Also, we did not assess for confounding variables such as previous exposure to cold water, which could bias our results. Finally, we assessed the study outcomes through self-reported measures, which could not be accurate for cognitive variables such as the executive functioning. Therefore, given these limitations, the results of this study should be interpreted with caution.

Future research should further explore the potentialities of combined training for maximising executive functioning and mood regulation effects by exploring the role at neurobiological level, for instance identifying the role of neurotransmitters during both activities. Moreover, considering that older adults tend to suffer from loneliness (44), it could be helpful to analyse the role of group vs. individual activities in the relationship between the type of training and the emotional/cognitive outcome.

The current study has several implications for the promotion of healthy living among middle-aged and older adults. First, the combination of mindfulness and cold water immersion could offer a non-pharmacological, low-cost, and accessible intervention for the support of psychological wellbeing. Moreover, the group dimensions of the activities might reduce loneliness and promote social connectedness among older adults. Finally, the intervention might specifically target the improvement of mood and executive functioning, whose decline may have negative consequences at individual (e.g., increased quality of life and mental health) and public health level (e.g., reduction of falls). Therefore, the implementation of this kind of interventions could significantly contribute to the reduction of the healthcare system burden.

## Data Availability

The raw data supporting the conclusions of this article will be made available by the authors, without undue reservation.
